# Phase-Based Cortical Synchrony Is Affected by Prematurity

**DOI:** 10.1093/cercor/bhab357

**Published:** 2021-10-20

**Authors:** Pauliina Yrjölä, Susanna Stjerna, J Matias Palva, Sampsa Vanhatalo, Anton Tokariev

**Affiliations:** Department of Clinical Neurophysiology, BABA Center, Children’s Hospital, Helsinki University Hospital and University of Helsinki, Helsinki, 00029 HUS, Finland; Department of Neuroscience and Biomedical Engineering, Aalto University, Helsinki, 00076 AALTO, Finland; Neuroscience Center, Helsinki Institute of Life Science, University of Helsinki, 00014 Helsinki, Finland; Department of Clinical Neurophysiology, BABA Center, Children’s Hospital, Helsinki University Hospital and University of Helsinki, Helsinki, 00029 HUS, Finland; Neuroscience Center, Helsinki Institute of Life Science, University of Helsinki, 00014 Helsinki, Finland; Division of Neuropsychology, HUS Neurocenter, Helsinki University Hospital and University of Helsinki, PL 340, 00029 HUS, Finland; Department of Neuroscience and Biomedical Engineering, Aalto University, Helsinki, 00076 AALTO, Finland; Neuroscience Center, Helsinki Institute of Life Science, University of Helsinki, 00014 Helsinki, Finland; Centre for Cognitive Neuroimaging, Institute of Neuroscience and Psychology, University of Glasgow, Glasgow G12 8QB, UK; Department of Clinical Neurophysiology, BABA Center, Children’s Hospital, Helsinki University Hospital and University of Helsinki, Helsinki, 00029 HUS, Finland; Neuroscience Center, Helsinki Institute of Life Science, University of Helsinki, 00014 Helsinki, Finland; Department of Clinical Neurophysiology, BABA Center, Children’s Hospital, Helsinki University Hospital and University of Helsinki, Helsinki, 00029 HUS, Finland; Neuroscience Center, Helsinki Institute of Life Science, University of Helsinki, 00014 Helsinki, Finland

**Keywords:** brain networks, neonatal EEG, neurodevelopment, phase coupling, preterm infant

## Abstract

Inter-areal synchronization by phase–phase correlations (PPCs) of cortical oscillations mediates many higher neurocognitive functions, which are often affected by prematurity, a globally prominent neurodevelopmental risk factor. Here, we used electroencephalography to examine brain-wide cortical PPC networks at term-equivalent age, comparing human infants after early prematurity to a cohort of healthy controls. We found that prematurity affected these networks in a sleep state-specific manner, and the differences between groups were also frequency-selective, involving brain-wide connections. The strength of synchronization in these networks was predictive of clinical outcomes in the preterm infants. These findings show that prematurity affects PPC networks in a clinically significant manner, suggesting early functional biomarkers of later neurodevelopmental compromise that may be used in clinical or translational studies after early neonatal adversity.

## Introduction

Approximately 10% of infants are born preterm, which inflicts lifelong disabilities in many key brain functions, including vision, learning, and language processing (WHO 2012; Johnson and Marlow 2017). Many of these functional abnormalities arise from the impacts that prematurity has on neuronal networks. Recent studies have demonstrated both structural (Batalle et al. 2017; Guo et al. 2017) and functional (Tóth et al. 2017; Tokariev, Roberts, et al. 2019a; Tokariev, Stjerna, et al. 2019b) effects of prematurity, some of which are shown to predict later neurodevelopmental outcomes.

Prematurity implies that the infants spend a part or all of their third trimester of gestation in an unnatural environment, ex utero*.* This time window is known to be characterized by the growth of brain networks driven by a combination of genetic and activity-dependent mechanisms (Luhmann et al. 2016; Molnár et al. 2020). The early cortical activity can be recorded with scalp electroencephalography (EEG) and consists of spontaneous intermittent bursts, which provide an early mechanism for inter-areal temporal correlations and define functional cortical networks (Vanhatalo and Kaila 2006). Therefore, the early cortical activity is a driver, guide, and biomarker of the development of brain networks.

The functional cortical networks can be characterized by quantifying relationships between phase or amplitude attributes of neural signals from distinct brain regions. Prior research on neonatal EEG (Omidvarnia et al. 2014; Tokariev, Roberts, et al. 2019a; Tokariev, Stjerna, et al. 2019b) have often focused on the amplitude–amplitude correlations that reflect co-modulation of overall neuronal activity and gross cortical excitability over periods of seconds (Palva and Palva 2011; Hipp et al. 2012; Engel et al. 2013; Tewarie et al. 2019). The other commonly used measure of neuronal interactions is phase–phase correlation (PPC) that is considered to reflect a spatiotemporally accurate mechanism of inter-areal communication. PPC is thought to arise from subsecond timing relationships in neuronal spiking (Womelsdorf et al. 2007; Palva and Palva 2011; Vidaurre et al. 2018), hence being able to support dynamic integration in neuronal ensembles underlying several higher-level brain functions (Bressler and Menon 2010; Palva and Palva 2011). Moreover, it is now well known that the brain operates concurrently at multiple frequencies, giving rise to multiplex networks shaped by concerted actions of different coupling mechanisms in several frequency bands (De Domenico et al. 2016; Siebenhühner et al. 2016).

Recent studies have suggested that PPC networks link to neurological performance (Tokariev, Stjerna, et al. 2019b) and early brain maturation (Gonzàlez et al. 2011; Van de Pol et al. 2018). However, the spatial and spectral extent of these findings, as well as their clinical correlates, have remained unclear. Here, we aimed to assess how the large-scale cortical PPC networks are affected by preterm birth of human infants. We analyzed EEG recordings from a large cohort of preterm and healthy control (HC) infants using an infant-specific source modeling-based analysis pipeline that allows noninvasive assessment of functional networks at the level of cortical sources. We asked whether prematurity leads to changes in the cortical networks that are linked to sleep state, brain area, or oscillation frequency. Moreover, we wanted to study if prematurity-related changes in cortical networks would have clinical significance, that is, be predictive of clinical neurological performance of the infants by the time of recording and/or later during childhood.

## Materials and Methods

**Figure 1:**
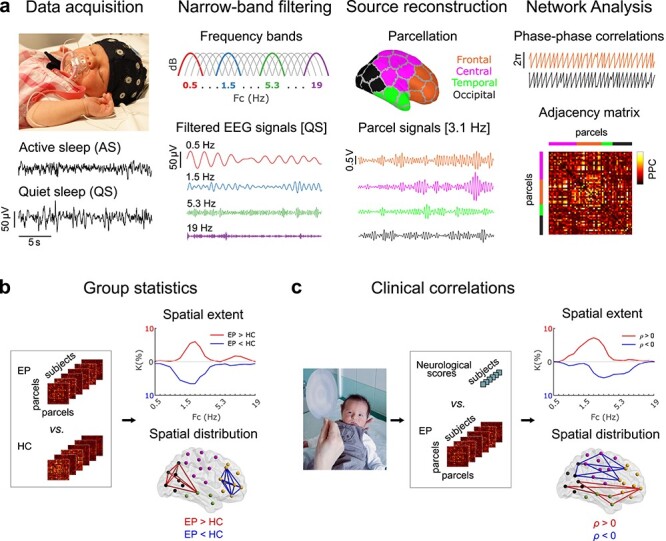
Outline of the study design and analyses. (*a*) EEG recordings of day-time sleep were acquired from EP and HC cohorts. The recordings were classified into AS and QS, and 5-min-long epochs were constructed for both sleep states. The selected epochs were filtered into 21 narrow frequency bands of semi-equal length on a logarithmic scale and converted to cortical source signals applying a realistic infant head model with 58 cortical parcels. Functional connectivity analysis was applied on the parcel signals by computing PPCs with the debiased weighted phase-lag index, yielding subject-specific adjacency matrices for both sleep states and all frequency bands. (*b*) Statistical group differences in connectivity strength were computed (Wilcoxon rank sum test) for both sleep states and each frequency band. The fraction of edges portraying significant differences (extent *K*) for 2 contrasts EP > HC (red) and EP < HC (blue) and the spatial distribution of the edges were then visualized. (*c*) Finally, correlations of PPC strengths to newborn neurological and 2-year neurocognitive assessment scores were investigated (Spearman correlation). The extent of edges with significant correlation between PPC and clinical outcomes (*K*) was computed and the spatial distribution of these edges visualized.

The overall analytical flow is shown in Figure 1 and described in detail in the following.

### Subjects

The dataset included *N* = 46 early preterm (EP) and *N* = 67 healthy controls (HC) infants. The gestational ages (GA) of the EP group (mean +/− standard deviation, SD) were 26.4 +/− 1.2 weeks and those of the HC group were 40.4 +/− 1.1 weeks. This dataset was collated from cohorts that have been published in previous studies (Rahkonen et al. 2013; Omidvarnia et al. 2014; Nevalainen et al. 2015; Tokariev, Videman, et al. 2016b; Leikos et al. 2019; Tokariev, Roberts, et al. 2019a; Tokariev, Stjerna, et al. 2019b). The study design was approved by the Ethics Committee of the Helsinki University Central Hospital and informed consent was obtained from a parent or guardian for each subject.

### E‌EG Recordings

Multichannel scalp EEG data were collected from both infant groups during day sleep. The requirement for the recording session was that each subject had to undergo 2 vigilance states: active sleep (AS) and quiet sleep (QS). EEG registration was performed using Waveguard caps with 19-28 sintered Ag/AgCl electrodes (ANT-Neuro, Berlin, Germany) located according to International 10-20 standard layout. Signals from both groups were recorded mostly with the NicOne EEG amplifier (Cardinal Healthcare, Ohio/Natus, Pleasanton, USA), but few EP subjects were recorded with the Cognitrace amplifier (ANT B.V., Enschede, the Netherlands). EEG recordings for both groups were performed at term-equivalent age of 41.4 ± 1.4 weeks CA (conceptional age, mean +/− SD). The original sampling frequency was 256 Hz or 500 Hz, but all data were resampled to 250 Hz when exporting them into European Data Format.

### Clinical Assessments

Newborn neurological assessments (Hammersmith Neonatal Neurological Examination [HNNE]; Dubowitz et al. 1999) were conducted on the EP and HC cohorts at term-equivalent age to quantify the infants’ neurodevelopment. The HNNE was selected due to its wide use in assessment of neurological behavior in multiple populations (Romeo et al. 2011; Venkata et al. 2020). The test comprises 6 separate domains of neurodevelopment: reflexes, movements, posture tonus, tone patterns, abnormal signs, and orientation and behavior. To render the outcomes suitable for studying associations with PPC networks, we performed dimensionality reduction by principal component analysis (PCA) as described earlier (Tokariev, Stjerna, et al. 2019b). PCA generates uncorrelated linear combinations of the input data, that is, principal components, by maximizing variance. We applied PCA to 3 individual tests (visual alertness, head raising in prone, and increased neck extensor tone), and then used first 2 components (C1 and C2, respectively) as combination scores. In the post hoc assessment, the resulting C1 was shown to correlate primarily with later motor performance (hereafter called “motor score”), whereas C2 was found to be associated with later cognitive and social performance (hereafter called “cognitive score”) (Tokariev, Stjerna, et al. 2019b). Higher scores represent higher cognitive/motor function.

Long-term neurocognitive follow-up assessment was performed only on the EP infants at 2 years of age, using Bayley Scales of Infant and Toddler Development (Bayley 2006) and the Griffiths Mental Developmental Scales (Huntley 1996). The neuropsychological follow-up data were not available for the HC infants. These neurocognitive assessment tests were chosen, because of their established, widespread clinical use plus a broad impact on lifelong neurocognitive performance and quality of life, hence supporting the translational potential of findings (Rogers and Hintz 2016; Hernandez 2018). While some other outcomes such as gross motor development or hearing may be sometimes affected in EP infants, prior studies have suggested that these are most likely modified by a host of individual and treatment interventions (Kilbride et al. 2018). As above, high scoring reflects higher neurocognitive performance.

### E‌EG Review and Preprocessing

Vigilance state assessment was performed through a combination of electrophysiological and behavioral measures, the latter observed using polygraphic channels (chin electromyogram, electrocardiogram, electrooculogram, and respiratory sensors; see also Omidvarnia et al. 2014, Tokariev, Roberts, et al. 2019a, Tokariev, Stjerna, et al. 2019b). EEG traces during AS exhibit continuous fluctuations, with an irregular respiration and occasional eye movements. Conversely, EEG during QS is characteristically discontinuous, with a regular respiration (André et al. 2010). We then selected 5-min long artifact-free EEG epochs from representative periods of AS and QS. To avoid transition periods between vigilance states, representative sleep epochs were selected from within well-established patterns of corresponding behavior and brain activity. Epochs that did not meet quality and length requirements were excluded from the data pool. As a result, we obtained 4 final groups: EP-AS (*N* = 46), HC-AS (*N* = 53), EP-QS (*N* = 42), and HC-QS (*N* = 66). For each subject, we selected the same *N* = 19 channels (Fp1, Fp2, F7, F3, Fz, F4, F8, T7, C3, Cz, C4, T8, P7, P3, Pz, P4, P8, O1, O2) to enable group-level analysis. All EEG signals were first prefiltered within the 0.15–45 Hz frequency range using a combination of high- and low-pass Butterworth filters of the seventh order. All filtering in this work were implemented offline and in forward–backward directions to compensate for phase delays introduced by infinite impulse response filters. Next, the EEG data were downsampled to a new sampling frequency, Fs = 100 Hz, and converted into average montage. Following our previous work (Tokariev, Roberts, et al. 2019a), we filtered the preprocessed EEG into 21 frequency bands of interest covering the physiologically relevant range 0.4–22 Hz (Vanhatalo et al. 2005). This narrow band approach was opted to allow sensitive scanning through the frequency domain and thereby capture networks that may not fall within predefined frequency bands. Band-pass filtering was implemented with pairs of low- and high-pass filters. The first central frequency (Fc) was set to 0.5 Hz and subsequent frequencies were computed as Fc(*i*) = 1.2 × Fc(*i*−1), where *i* is the number of the frequency band. Cut-off frequencies for each band were taken as 0.85 × Fc and 1.15 × Fc correspondingly. This approach leads to 50% overlapping frequency bands of semi-equal width in the logarithmic scale.

### Computation of Cortical Signals

Band-pass filtered EEG was further source reconstructed to allow better spatial separation of cortical activities using a realistic infant head model (Tokariev, Stjerna, et al. 2019b) and dynamic statistical parametric mapping (Dale et al. 2000). Theoretically, source reconstruction could be marginally more accurate by using individual head models based on individual magnetic resonance imaging (MRI) images taken near the time of EEG recording, especially in the preterm infants where brain structure might somewhat deviate from an average population. This would also need digitization of the EEG coordinates in each recorded individual. However, obtaining such MRI data and electrode digitizations is not technically or ethically possible in human infants, hence we opted to use the common MRI template from one infant as the average head model. As the source space, we used normal to cortical surface (at term age) dipoles of fixed orientation (*N* = 8014). The scalp and inner/outer skull shells were segmented from MRI data from healthy full-term infant. Following previous studies (Despotovic et al. 2013; Odabaee et al. 2014; Tokariev, Vanhatalo, et al. 2016a), tissue conductivities were set to: 0.43 S/m for scalp, 1.79 S/m for intracranial volume, and 0.2 S/m for skull. Finally, cortical sources were clustered into *N* = 58 parcels according to the scheme optimized for infant EEG. Cortical signals representing neural activity of each parcel were computed as the weighted mean of source signals belonging to the host parcels (Tokariev, Stjerna, et al. 2019b).

### Computation of Functional Connectivity

To estimate functional connectivity, we computed PPCs between all pairs of parcels using the debiased weighted phase-lag index, (Vinck et al. 2011). We opted to use this metric because of its robustness to artificial interactions caused by volume conduction (Palva and Palva 2012; Palva et al. 2018). Connectivity was estimated using whole 5-min-long epochs, at 21 frequency bands and for both vigilance states (AS and QS). This led to a set of 58 × 58 PPC matrices for each subject, accumulating to a total of 1653 pairwise connections. Then, we assessed how reliably the PPC interaction can be captured in each individual parcel pair. This was done by forward/inverse simulations as detailed before (Tokariev, Stjerna, et al. 2019b). The EEG-derived connectivity matrices were corrected with a binary “fidelity” mask, which defines the reliable connections when using the given electrode layout. This procedure aims to improve the reliability of cortical-level network estimation from a suboptimal number of recording electrodes, which are usually used in clinical recordings as discussed in Tokariev, Vanhatalo, et al. (2016a).

### Network Analysis

To test network differences between EP and HC cohorts, we applied the Wilcoxon rank sum test (2 one-tailed tests, α = 0.01) in an edge-by-edge manner with defined directions (EP > HC and EP < HC). This was done for each frequency band and for each sleep state separately. As a result of such edgewise scanning, we obtained matrices of *P*-values corresponding to the network connections and computed the ratio (*K*) of edges which were significantly different between the cohorts (Palva et al. 2010). To estimate the potential number of false discoveries in the frequency-specific group contrasts, we employed the Storey–Tibshirani adaptive FDR method using *q* = 0.01 with respect to the size of the whole network (i.e., up to *N* = 1128 × 0.01 = 11 connections in each network were classified as potential false discoveries), (Storey and Tibshirani 2003; Puoliväli et al. 2020). Effect size for the significantly different patterns was computed as a module of the mean of rank-biserial correlation values for corresponding connections. The influence of age differences on network strength was investigated by correlating the global mean connectivity strength with age for each group per frequency and sleep state (Spearman correlation, two-tailed test, α-level 0.05). *P*-values of both sleep states were pooled together separately for each group and controlled for multiple comparisons by the Benjamini–Hochberg procedure (Benjamini and Hochberg 1995).

Parallel to the primary analysis, a cross-check for statistical group comparison was performed using network-based statistics (NBS) (Zalesky et al. 2010) separately for the 21 frequency bands and 2 sleep states using 2 one-tailed tests (EP > HC and EP < HC). NBS is a multiple comparisons method designed specifically for network analysis. It assumes that connections reflecting true effects are interconnected into networks encompassing more than a single connection. The connected components are defined in a topological space, in contrary to other cluster-based methods, which use a physical space (Genovese et al. 2002; Zalesky et al. 2010). The initial threshold for the *t*-statistic was set to 2.5, followed by the post hoc permutation test to correct the family-wise error rate (5000 permutations, α = 0.05).

### Clinical Correlation

The connectivity strength of each edge across the infant group was correlated with the corresponding standardized neurological assessments at term-equivalent age and with the corresponding neurocognitive performance scores at 2 years of age (Spearman, two-tailed test with α-level 0.05) with CA as a covariate. We computed the fraction of edges showing significant clinical correlation (*K*) for each frequency band and sleep state. Only the EP cohort had performance scores tested at 2 years of age. Some subjects had missing clinical scores, rendering the number of subjects used for each correlation to: C1: EP-AS (*N* = 39), EP-QS (*N* = 36), HC-AS (*N* = 30), HC-QS (*N* = 51); C2: EP-AS (*N* = 39), EP-QS (*N* = 36), HC-AS (*N* = 40), HC-QS (*N* = 51); Griffiths Visual: EP-AS (*N* = 35), EP-QS (*N* = 32); Griffiths Motor: EP-AS (*N* = 39), EP-QS (*N* = 36); Bayley Cognitive: EP-AS (*N* = 32), EP-QS (*N* = 30); Bayley Language comprehension: EP-AS (*N* = 30), EP-QS (*N* = 28). Multiple comparisons correction was implemented with the Storey–Tibshirani adaptive FDR with *q* = 0.05 (i.e., 2.5% of the positive and negative correlations separately were classified as potential false discoveries). The Spearman *ρ*-value was used for estimating effect size: It was computed for all significant edges and averaged across the full network.

### Analysis Software

Source reconstruction was conducted using the Brainstorm (Tadel et al. 2011; https://neuroimage.usc.edu/brainstorm/Introduction) and the openMEEG (Gramfort et al. 2010; https://openmeeg.github.io/) software packages. Analyses were performed with Matlab R2020a (MathWorks, Natick, MA) and NBS Connectome (Zalesky et al. 2010), (https://www.nitrc.org/projects/nbs/), and the visualization of brain networks was carried out with BrainNet Viewer (Xia et al. 2013; https://www.nitrc.org/projects/bnv/).

The Matlab script implementing the network analyses of group differences and clinical correlation can be found at https://github.com/pauliina-yrjola/Preterm-Phase.

## Results

### P‌PC Networks Are Affected by Prematurity in a Frequency-Specific Manner

**Figure 2:**
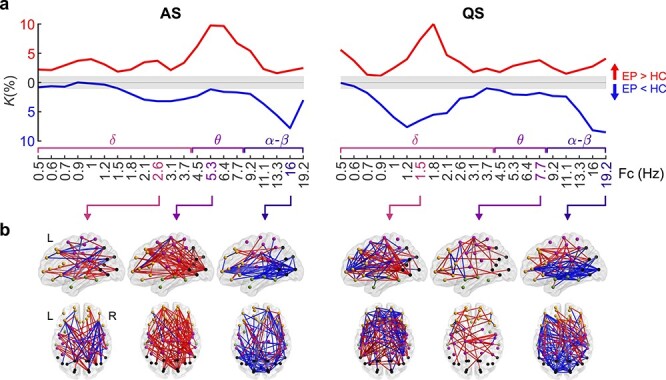
Effects of prematurity on cortical PPC networks. (*a*) Network density (*K*) of significant PPC group differences (2 one-tailed Wilcoxon rank-sum tests, α = 0.01) during AS (left) and QS (right) as a function of frequency. Networks that are stronger in EP (EP > HC) are shown in red, whereas networks with suppressed connectivity in EP (EP < HC) are presented in blue. The gray shaded area depicts the boundaries of the *q*-level showing the potential level of false discoveries (*q* = 0.01). The data presented in the figure are provided in Figure 2—source data 1 and matrices of the *P*-values and effect sizes of all networks in Figure 2—source data 2. (*b*) Spatial visualizations present PPC network comparisons at the frequencies with the most extensive group differences. The color coding of the networks (red, blue) corresponds to that of (*a*).

To evaluate the impact of prematurity on cortical networks as a function of frequency, we estimated the extent of significant PPC network differences between EP and HC groups at each specific frequency. We described the extent of patterns reflecting group differences as a fraction (*K*) and visualized the spatial distribution of these patterns (Fig. 2).

We found broad sleep- and frequency-specific differences in PPC networks between EP and HC infants (Fig. 2). During AS, the most extensive group differences were observed within the theta frequency band (peak at Fc = 5.3 Hz; Fig. 2*a*) with stronger connections in the EP group (*K* = 10%, *P* < 0.01, *q* = 0.01) that were uniformly distributed over the whole cortex (Fig. 2*b*). Smaller subnetworks (*K* = 2–4%, *P* < 0.01, *q* = 0.01) over multiple cortical regions were found at delta frequencies, showing different edges of the network with increased (1.8–3.1 Hz) and decreased (1.5–4.5 Hz) connectivity strengths in the EP infants. During QS, the most prominent group differences were within the delta band: The EP infants exhibited stronger connectivity (*K* = 10% at 1.8 Hz, *P* < 0.01, *q* = 0.01) in mostly long-range inter-areal connections, while there were weaker short-range connections (*K* = 8% at 1.2 Hz, *P* < 0.01, *q* = 0.01) within the frontal lobe and a few projections to the parietal and occipital lobes. Networks at alpha and beta frequencies were suppressed in the EP infants during both sleep states, and they involved dense basal connections linking occipital cortices to frontal and temporal areas. All findings were characterized by gradual changes relative to frequency band, as shown in Figure 2—figure supplement 1.

The possible systematic effect of age on PPC values was checked, yet no significant correlations were found between mean connectivity strength and age at any frequency (Spearman, two-tailed test, α = 0.05, Benjamini–Hochberg correction). Effect sizes, computed by the rank-biserial correlation over each significant network, are presented in Figure 2—figure supplement 2. We also validated the results with an alternative analysis using NBS (Zalesky et al. 2010), with 2 one-tailed tests (for details, see Methods), and found similar spectral and spatial patterns in group comparisons (Figure 2—figure supplement 3). The findings together suggest that exposure to prematurity affects the organization of cortical PPC networks at multiple oscillatory frequencies, yet the target network is highly dependent on frequency.

### Connectivity Strength Correlates with Neurological Performance in Preterm Infants

**Figure 3:**
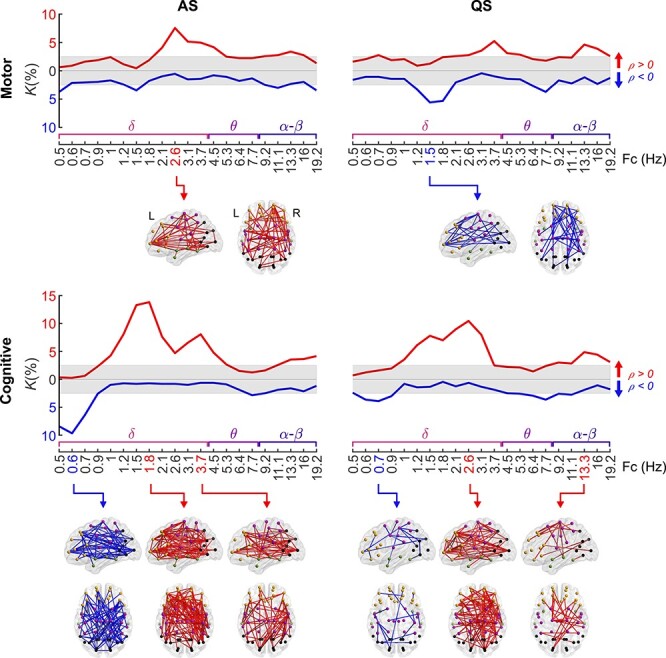
PPC networks of ex-preterm infants at term age predict neurological outcome. Density (*K*) of PPC patterns that associate to neurological scores linked to later motor and cognitive performance (Spearman, two-tailed test with CA as a covariate, α = 0.05) as a function of frequency. The gray shaded area depicts the FDR boundaries (*q* = 0.05). The opaque brains show the spatial distributions of networks taken at the most characteristic peaks of the density curves. Red coloring pictures networks with positive correlation (*ρ* > 0), while blue coloring shows negatively correlated connections (*ρ* < 0) in both the graphs and 3-dimensional plots. The graph data are provided in Figure 3—source data 1 and the full *P*-value and effect size matrices in Figure 3—source data 2.

Next, we studied how the strength of cortical PPC networks correlates to neurological performance at the time of newborn EEG recordings. To this end, we correlated the connectivity strengths of each PPC network connection (*N* = 1128) of the EP group to the neurological performance of the corresponding infants, assessed using compound scores which are associated with later motor and cognitive outcomes (Tokariev, Stjerna, et al. 2019b). We quantified the extent of significantly correlated connections using a density measure (*K*) and visualized the networks showing broad spatial effects (Fig. 3).

The cognitive score was positively correlated with an extensive pattern at higher delta frequencies in both sleep states (AS: 1.2–4.5 Hz, *K* = 5–14%, QS: 1.2–3.1 Hz, *K* = 6–10%, *P* < 0.05, *q* = 0.05). The corresponding spatial patterns incorporated broad networks linking multiple distal areas. In contrast, the motor score showed only mildly elevated density, or small networks, with positive correlation at 2.6–4.5 Hz during AS (*K* = 4–8%, *P* < 0.05, *q* = 0.05) and a negative correlation at 1.5–1.8 Hz during QS (*K* = 5–6%, *P* < 0.05, *q* = 0.05). Effect sizes (mean of Spearman *ρ* over the positive and negative correlation networks separately) are depicted in Figure 3—figure supplement 1. A comparable analysis for HCs (Figure 3—figure supplement 2) showed only a few negative correlations between edge strength and neurological scores. The spatial distributions for all investigated frequency bands are presented in Figure 3—figure supplement 3 for the motor score and Figure 3—figure supplement 4 for the cognitive score.

These findings together suggest that the relationship between cortical networks and neurological performance is affected by prematurity. The EP infants exhibit brain-wide relationships between cortical networks and neurological performance, which is not seen in the HC infants.

### Correlation of Functional Connectivity and Neurological Performance Extends to Long-Term Neurocognitive Outcomes

**Figure 4:**
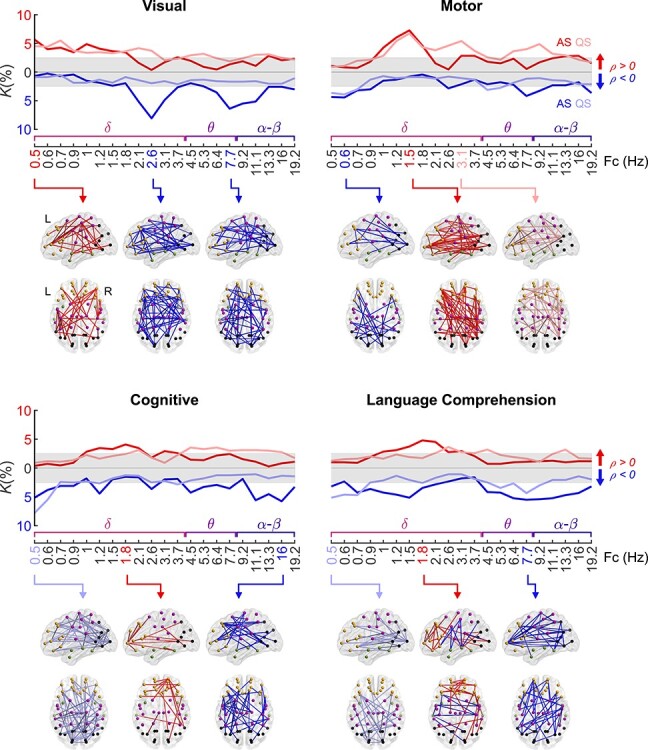
Correlation of PPC network strength to 2-year neurocognition. The upper graphs show the frequency-wise summary of the proportion of network edges (*K*) that show a significant correlation between PPC strength and the given neurocognitive performance score (Spearman, two-tailed test with CA as a covariate, α = 0.05). The FDR (*q* = 0.05) boundaries are depicted as a gray shaded area. The strongest peaks in these plots were selected for the 3-dimensional visualizations of networks as indicated with arrows. Color coding represents the sign of correlation (red: *ρ* > 0, blue *ρ* < 0) and hues represent sleep states (dark: AS, light: QS) in the graphs and the spatial visualizations. The data displayed in the curves are provided in Figure 4—source data 1 and the *P*-value and effect size matrices from which the graphs were created in Figure 4—source data 2.

Finally, we examined the relation of PPC networks to long-term neurocognitive development, assessed at 2 years of age using standardized Bayley (Bayley 2006) and Griffiths (Huntley 1996) scores. Akin to our analysis above on newborn clinical performance, we correlated the strength of individual connections in the PPC networks of the EP infants to their clinical outcome measures at 2 years of age. Most of the significant correlations emerged for visual, motor, cognitive, and language comprehension scores at lower frequencies (Fig. 4), forming mostly spatially constrained patterns.

Visual scores correlated negatively with PPC during AS with peaks at Fc = 2.6 Hz and Fc = 7.7 Hz (*K* = 6–8%, *P* < 0.05, *q* = 0.05). The correlation networks incorporated multiple areas over the whole cortex. A small set of positively correlating connections was found at Fc = 0.5 Hz during both sleep states (*K* = 5–6%, *P* < 0.05, *q* = 0.05).

Motor scores featured a prominent positive correlation during both sleep states at Fc = 1.5 Hz (*K =* 7%, *P* < 0.05, *q* = 0.05) with a dense spatial distribution over several cortical regions. We also found a somewhat smaller extent network with a positive correlation to motor score during QS at a slightly higher frequency (Fc = 3.1 Hz; *K* = 5%, *P* < 0.05, *q* = 0.05). Finally, a subset of mostly occipital interhemispheric connections showed negative correlation to motor scores during both sleep states at the lowest frequencies (Fc = 0.5, 0.6 Hz; *K* = 4%, *P* < 0.05, *q* = 0.05).

The cognitive performance score showed negative correlations at low (AS and QS; Fc = 0.5 Hz; *K* = 5–8%, *P* < 0.05, *q* = 0.05) and high frequencies (AS only; Fc = 16 Hz; *K* = 6%, *P* < 0.05, *q* = 0.05). A few connections displayed positive correlations with cognitive performance during AS at Fc = 1.8 Hz (*K* = 4%, *P* < 0.05, *q* = 0.05), linking frontal nodes to other regions.

Language comprehension correlated to PPC networks in AS (peak at Fc = 1.8 Hz, *K* = 5%, *P* < 0.05, *q* = 0.05), involving connections between frontal and temporal regions. A negative correlation between PPC strength and language comprehension scores was present in long-range connections at lower frequencies during QS (Fc = 0.5 Hz, *K* = 5%, *P* < 0.05, *q* = 0.05), as well as a brain-wide network at mid-frequencies during AS (Fc = 7.7 Hz, *K* = 4%, *P* < 0.05, *q* = 0.05).

**Figure 5:**
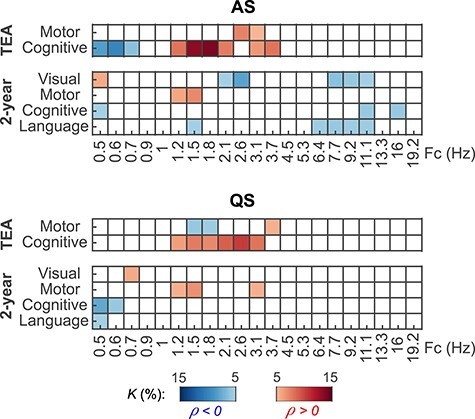
Summary of main correlations. The graphs show a summary of network density (*K*) at each frequency for correlations to outcome measures at term-equivalent age (TEA) and 2 years of age (Spearman, two-tailed test with CA as a covariate, α = 0.05). Positive correlations (*ρ* > 0) are shown in shades of red and negative correlations (*ρ* < 0) in shades of blue. *K* is thresholded at 5% to highlight the most salient correlations. The data presented in the figure are provided in Figure 5—source data 1.

Effect sizes were computed as the mean of Spearman *ρ* of the positive and negative networks separately and are presented as a function of frequency in Figure 4—figure supplement 1. The spatial distributions at all investigated frequency ranges are shown in Figure 4—figure supplement 2–5. A summary of the most prominent correlations is presented in Figure 5.

## Discussion

Our study shows that spontaneous cortical activity in the human infants exhibits large-scale PPC structures, which are spectrally and spatially selective and co-vary with vigilance states. Moreover, we show that the globally most significant clinical risk factor, preterm birth (WHO 2012), leads to frequency-selective changes in these networks and that the newborn networks of ex-preterm infants also correlate to neurocognitive performance. Our work employed novel realistic cortical source reconstruction and independent parallel analyses to validate the results on clinical network correlations. Our findings are broadly consistent with recent work on adults showing that multiple frequency-specific PPC networks coexist (De Domenico et al. 2016; Siebenhühner et al. 2016; Yu et al. 2017; Vidaurre et al. 2018) and correlate with normal and pathological behaviors (Siebenhühner et al. 2016; Yu et al. 2017). Our work extends prior studies reporting prematurity effects on the temporally loose amplitude correlations (Omidvarnia et al. 2014; Tokariev, Roberts, et al. 2019a); here, we provide evidence that the cortico-cortical interactions in newborn infants are already accurate enough to give rise to spectrally and spatially specific PPC structures, and pathological effects therein.

It has recently become clear that brain function relies on several co-existing frequency-specific PPC networks, which are reported to show temporal dynamics between awake states in adults (Siebenhühner et al. 2016; Vidaurre et al. 2018) or between sleep states in the neonatal studies (Tokariev, Videman, et al. 2016b; Tokariev, Stjerna, et al. 2019b). Here, we show that medical adversities can affect these PPC networks in a selective manner, at preferential frequencies, and with preferential spatial distributions, as well as differing between vigilance states. For instance, prematurity caused an increase in middle frequency PPC in long-range connections throughout the brain, while the changes in higher frequencies were more localized in the middle and long-range connections in the basal brain areas. These effects were more pronounced during AS for the middle frequencies, while high-frequency findings were essentially similar between sleep states. The findings are compatible with a notion that the functional significance of frequency-specific PPC networks depends on their context, the brain state, in addition to their given frequency. The previously described diffuse and extensive white matter abnormalities after prematurity (Dimitrova et al. 2020) may provide a straightforward histological underpinning for the changes. Since PPC is known to require high temporal precision in neuronal communication (Womelsdorf et al. 2007; Palva and Palva 2012), any compromise in neuronal conduction velocities due to myelination changes would readily explain the observed decrease in higher frequency PPC networks. The major white matter tracts are presumably often affected (Kostović et al. 2014; Vasung et al. 2016; Vasung et al. 2017). The frontal involvement in many found PPC effects may be explained by the way how frontal regions develop late (Mrzljak et al. 1992; Vasung et al. 2017) and establish their cortico-cortical pathways via the subcortical cross-roads, a key site in early developmental brain insults (Judas et al. 2005; Vasung et al. 2010). The well-established transient emergence of exuberant connections during early development (Kostović et al. 2019; Innocenti 2020), followed by their later activity-dependent pruning, may offer a histological underpinning for the observed higher PPC levels in the long-range connections of the preterm infants. The high inter-individual variability of structural effects, however, does not allow an accurate prediction of the spatial topography in the EEG-based network change. It is also commonly observed that the network changes may be more diffuse than their associated structural changes (Zhang et al. 2021). A full understanding of the present findings would require combining the known structural connectome (Kostović et al. 2014; Vasung et al. 2016; Vasung et al. 2011) to the spatially and spectrally selective PPC network topologies described in our work. Recent work in adults has suggested that functional and structural networks may share common skeletons (Finger et al. 2016; Mostame and Sadaghiani, 2020). The high dimensionality in the newborn EEG-based network data, however, precludes a straightforward combination with anatomical connectome templates.

Our present findings extend the long-held clinical tradition where QS is considered to be the most sensitive state in disclosing effects of prematurity in the EEG records. In the clinical visual review, the EEG signal is considered to exhibit dysmature/immature features (Lombroso 1979; Tharp 1990), and the most robust feature is augmented “interhemispheric asynchrony,” or temporal nonoverlap between cortical bursting (Räsänen et al. 2013; Koolen et al. 2014). While the clinically perceived interhemispheric asynchrony considers QS and amplitude correlations only, here we show that robust prematurity effects are also seen in the PPC networks, and they are clear during AS. Moreover, the functional significance of the PPC network during AS is shown by their pronounced correlations to subject-level clinical performance.

The strength of PPC connectivity in several brain-wide subnetworks was found to correlate to infants’ neurological performance at term age, which extends prior reports on clinical correlations to frontally connected delta frequency networks (Tokariev, Stjerna, et al. 2019b). Clinical correlations were clearly widest for the composite score, which emphasizes features of newborn performance that preempt later cognitive development (Tokariev, Stjerna, et al. 2019b). Comparison to neurocognitive performance at 2 years of age showed also several albeit smaller PPC subnetworks with significant correlations.

While our findings suggest clinically meaningful functions for the herein characterized PPC networks in the EP infants, it was somewhat unexpected that comparable correlations were not found in the group of HC infants. That observation suggests an altered relationship between PPC networks and neurocognitive phenotypes in prematurely born infants, which calls for a reasonable mechanistic explanation. It is possible that the network–phenotype relationship becomes amplified in the preterm cohort that is known to exhibit considerable variation in their histological maturation (Dimitrova et al. 2020). Prior studies have shown brain-wide effects of prematurity on the histological structures of white matter tracts (Dimitrova et al. 2020; Kostović et al. 2014; Vasung et al. 2016; Vasung et al. 2011), and these changes were shown to correlate with several characteristics of newborn or later neurocognitive performance (Stjerna et al. 2015; Vollmer et al. 2017; Girault et al. 2019; Toulmin et al. 2020). An alternative mechanism is that the effects found in EP infants reflect a transient network immaturity (a.k.a. dysmaturity, Lombroso 1979; Tharp 1990) that would catch up during later development. Testing this hypothesis would need repeated EEG network studies in the EP infants near term-equivalent age, as well as a relevant control group, to show a developmental catch up in the PPC networks (Tokariev, Videman, et al. 2016b) or in other functional brain age (Stevenson et al. 2020).

The present results suggest a clinically meaningful effect on PPC networks that could potentially serve as a functional biomarker to benchmark early therapeutic interventions (Ewen et al. 2019; Sahin et al. 2020). Such biomarkers could be constructed from, for example, the strength of coupling in the frequency-specific subnetworks identified in our work. Our current study needs to be considered as observational work that identified putative analysis pipelines and network markers. Future prospective studies on larger cohorts are needed to reduce the complexity of analytic procedures and to validate the consistency of findings. Our sample size estimation, based on the effect sizes observed in this work, suggests that a cohort of 113 infants is required for such a study (α = 0.05, β = 0.1, see Hulley et al. 2013). Such work would also define the perceived added value of network assessment from the perspective of monitoring early neurodevelopment and benchmarking early therapeutic interventions. In addition, these network effects may offer a unique translational bridge as a functional benchmark between preclinical models of prematurity and the human preterm infants.

## Notes

We want to thank the infants’ parents and research nurses for participation and technical assistance in the study. Dr Aulikki Lano, Dr Marjo Metsäranta and Prof. Sture Andersson are acknowledged for their efforts in the previously published study cohorts that provided some data to this work. *Conflict of Interest*: None declared.

## Funding

Finnish Pediatric Foundation, the Finnish Academy (313242, 288220, 321235), Juselius Foundation, Aivosäätiö, Neuroscience Center at University of Helsinki, and Helsinki University Central Hospital.

## Supplementary Material

Supplementary_figures_bhab357
