# Supplementary material for: Phase-Based Cortical Synchrony Is Affected by Prematurity
Source: Cereb Cortex. 2021 Oct 20;32(10):2265–76. doi: 10.1093/cercor/bhab357 (PMC9113310; doi:10.1093/cercor/bhab357)
Supplement: Supplementary_figures_bhab357 [file supplementary_figures_bhab357.pdf]

Supplementary Figures

Phase-based cortical synchrony is affected by prematurity

Pauliina Yrjölä[1,2,3], Susanna Stjerna[1,3], Matias Palva[2,3,4], Sampsa Vanhatalo[1,3]* & Anton Tokariev[1,3]*

* These authors contributed equally

1) BABA center, Department of Clinical Neurophysiology, Children's Hospital, Helsinki University Hospital and University of Helsinki, Helsinki, Finland
2) Department of Neuroscience and Biomedical Engineering, Aalto University, Helsinki, Finland
3) Neuroscience center, Helsinki Institute of Life Science, University of Helsinki, Helsinki, Finland
4) Centre for Cognitive Neuroimaging, Institute of Neuroscience and Psychology, University of Glasgow, United Kingdom

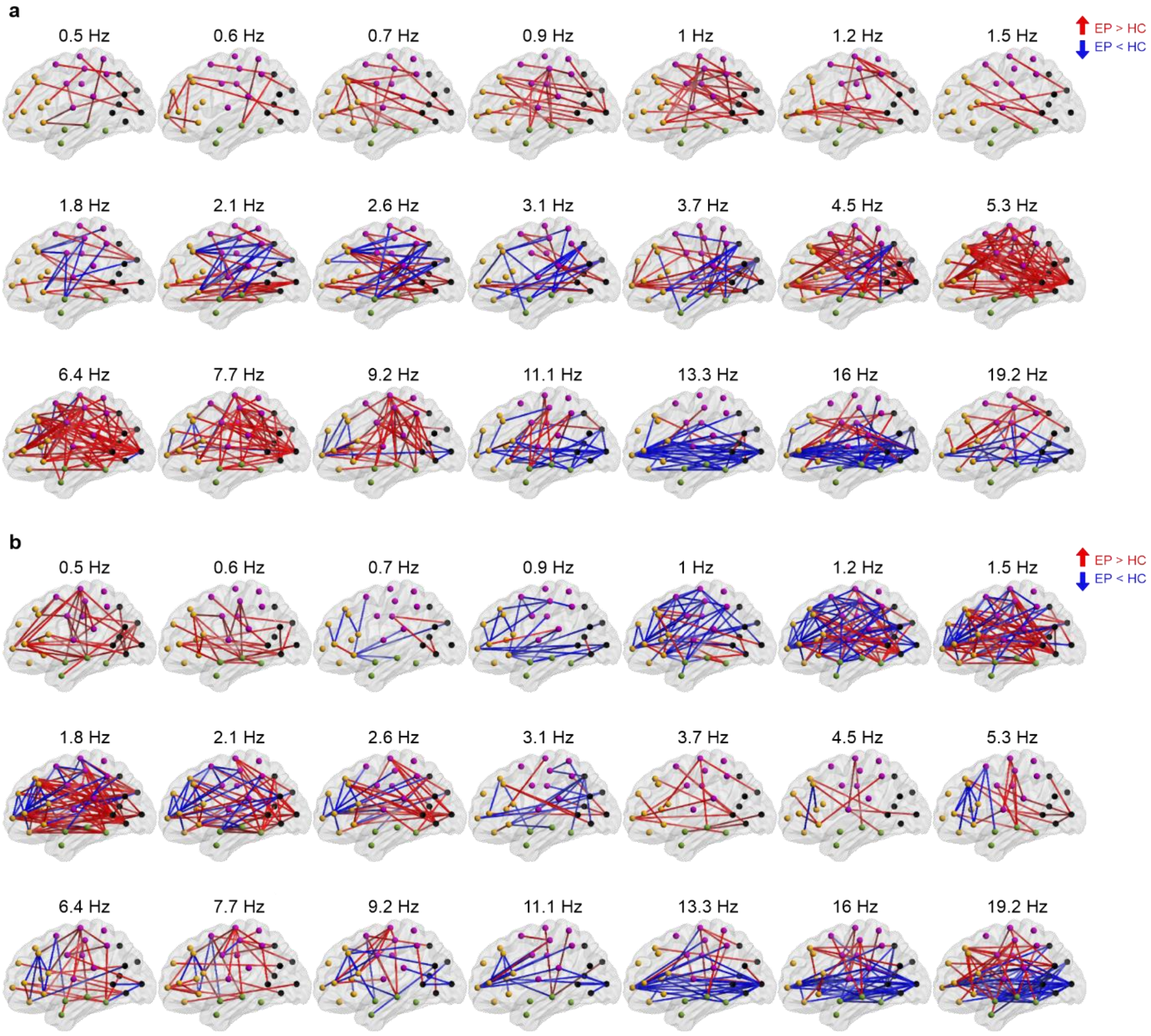

Figure 2—figure supplement 1. **Effects of prematurity on cortical PPC networks modulate over frequency.** Spatial visualizations of the group difference networks obtained from network density measurements (Figure 1, two one-tailed Wilcoxon rank sum tests, α = 0.01) over all frequency bands in AS (a) and QS (b). Only the edges which passed FDR correction (q = 0.01) are shown. Red networks display connections of increased connectivity in EP (EP > HC) and blue networks reduced connectivity in EP (EP < HC).

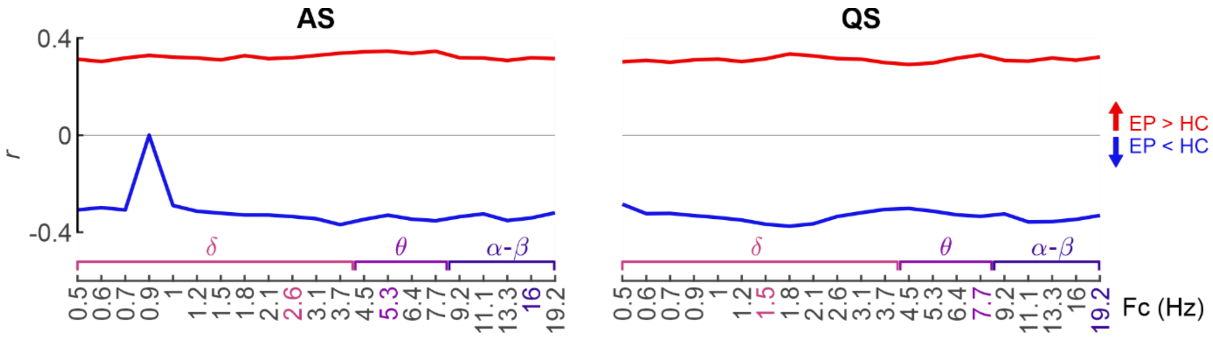

Figure 2—figure supplement 2. **Effect size of the statistical group differences on cortical PPC networks.** The effect size, computed by the mean rank-biserial correlation (*r*) of the significant networks shown in Figure 1 (two one-tailed Wilcoxon rank sum tests, α = 0.01) during active sleep (AS, left) and quiet sleep (QS, right), as a function of frequency. Networks with increased connectivity strength in EP (EP > HC) are shown in red, and networks with decreased connectivity in EP (EP < HC) are displayed in blue. The coloured numbers depict the frequencies with the most extensive group differences (Figure 2).

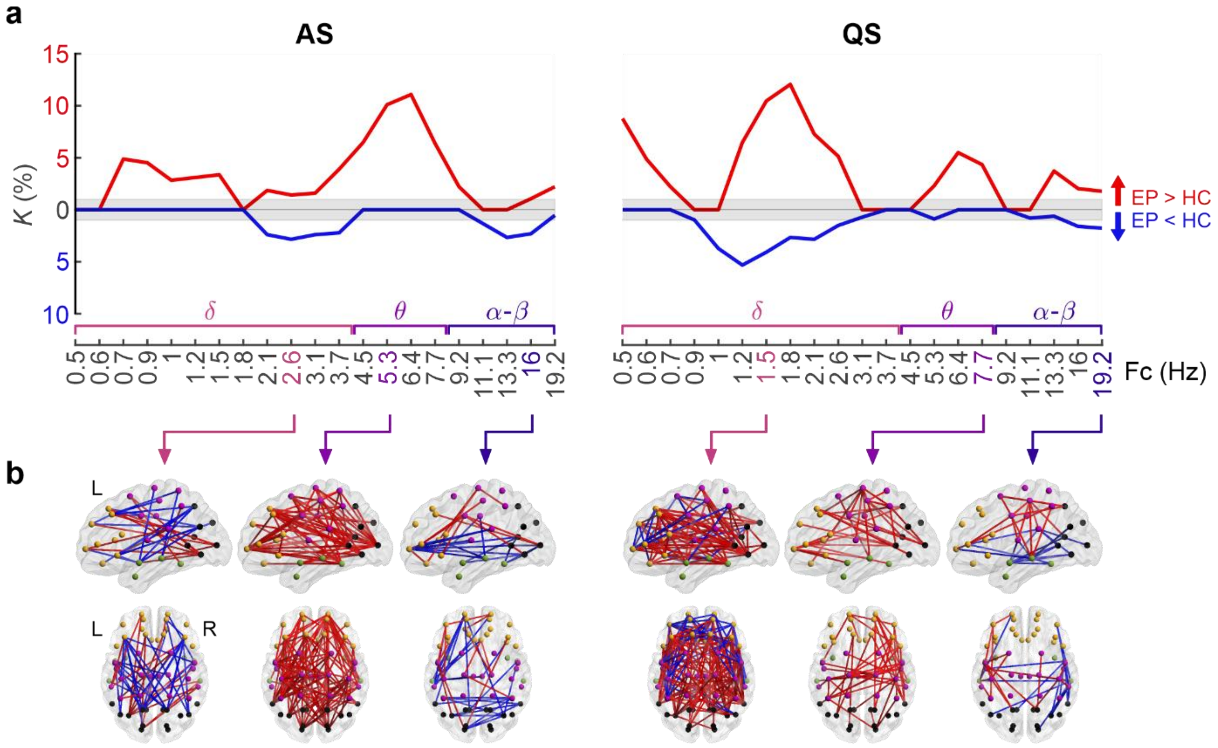

Figure 2—figure supplement 3. **Effects of prematurity on cortical PPC networks replicated with an independent additional analysis. (a)** Network density (*K*) of PPC group difference networks computed with NBS Connectome (two one-tailed tests, threshold 2.5, significance level α = 0.05, 5000 permutations) is shown for AS (left) and QS (right) as a function of frequency bands, denoted by their central frequencies. The directions of difference (EP > HC or EP < HC) are indicated in red and blue, respectively. **(b)** 3-dimensional visualizations present the spatial distribution of group difference networks at the frequencies selected from Figure 1. The direction of difference is indicated as in (a). The network extent curves and spatial distributions both resemble closely those of the primary analysis and thus give them support.

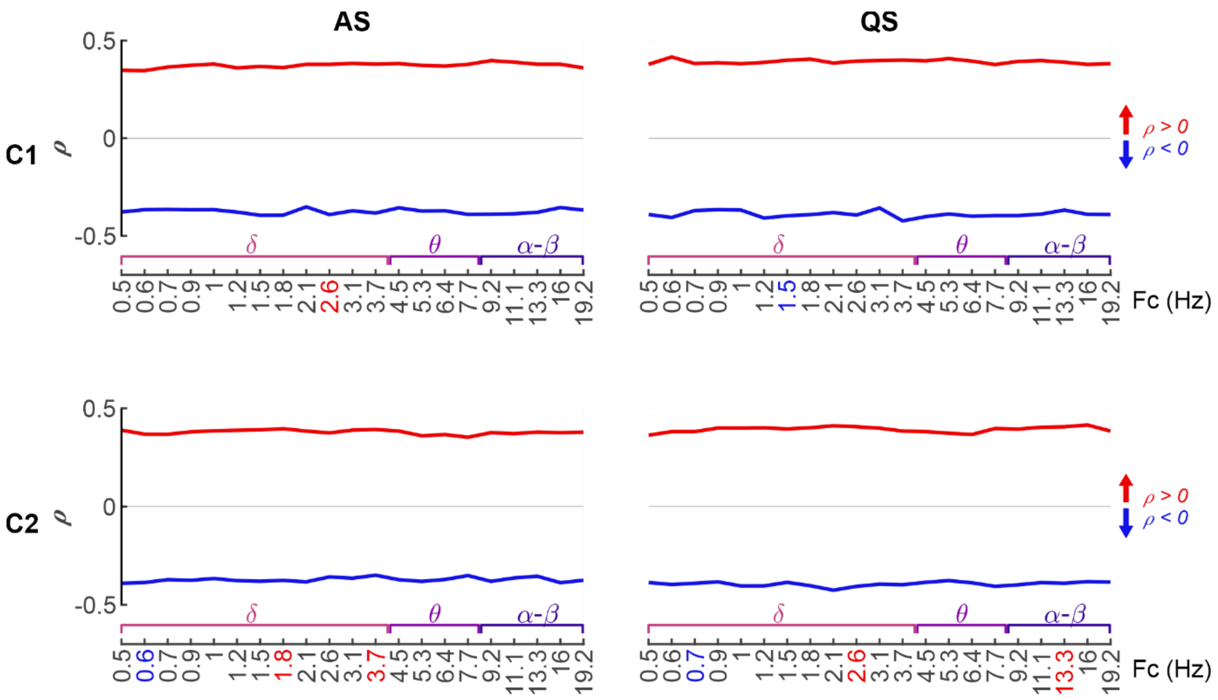

Figure 3—figure supplement 1. **Effect size of PPC networks depicting clinical correlation.** The effect size, acquired from the mean Spearman (two-tailed test, with conceptional age as a covariate, and α = 0.05) *ρ*-value of the positive (*ρ* ≥ 0, red) and negative (*ρ* < 0, blue) networks, as a function of frequency band. The results are presented for active sleep (AS, left) and quiet sleep (QS, right), as well as for the neurological outcome scores C1 (above) and C2 (below). The coloured numbers depict the frequencies with the most extensive correlations (Figure 3).

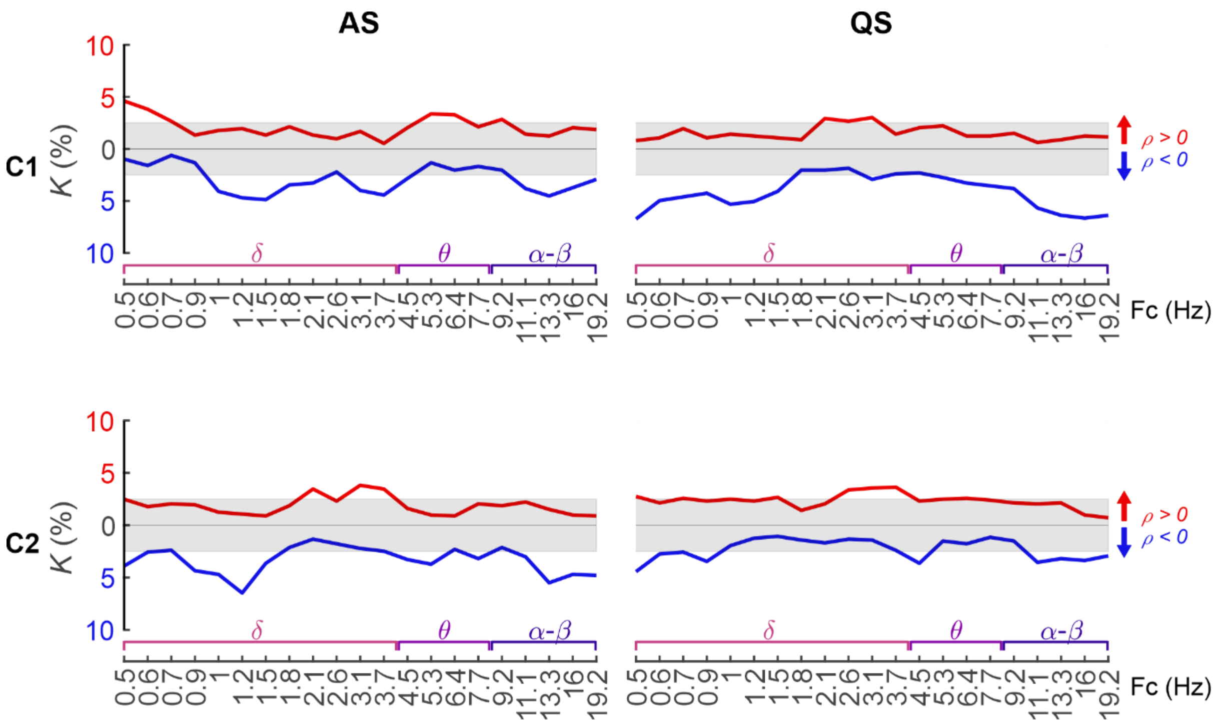

Figure 3—figure supplement 2. **Absence of correlation between cortical PPC strengths and early neurological performance in healthy controls.** Network density (*K*) of PPC correlation related to the neurological assessment scores C1 and C2 as a function of frequency band in the HC cohort (Spearman, two-tailed test, with conceptional age as a covariate, and $\alpha = 0.05$). The FDR ($q = 0.05$) boundaries are depicted as a grey shaded area. Colour coding represents the polarity of the correlation (red: $\rho \geq 0$, blue $\rho < 0$). Analysis of the HC group shows an absence of wider network patterns that would correlate positively to either of the neurological scores. There are some networks at low frequencies with negative correlation to C1 and C2 during AS (peak at Fc = 1.2–1.5 Hz) and some networks at low and high frequencies with negative correlation to C1 during QS (peaks at Fc = 0.5 Hz and Fc = 16 Hz).

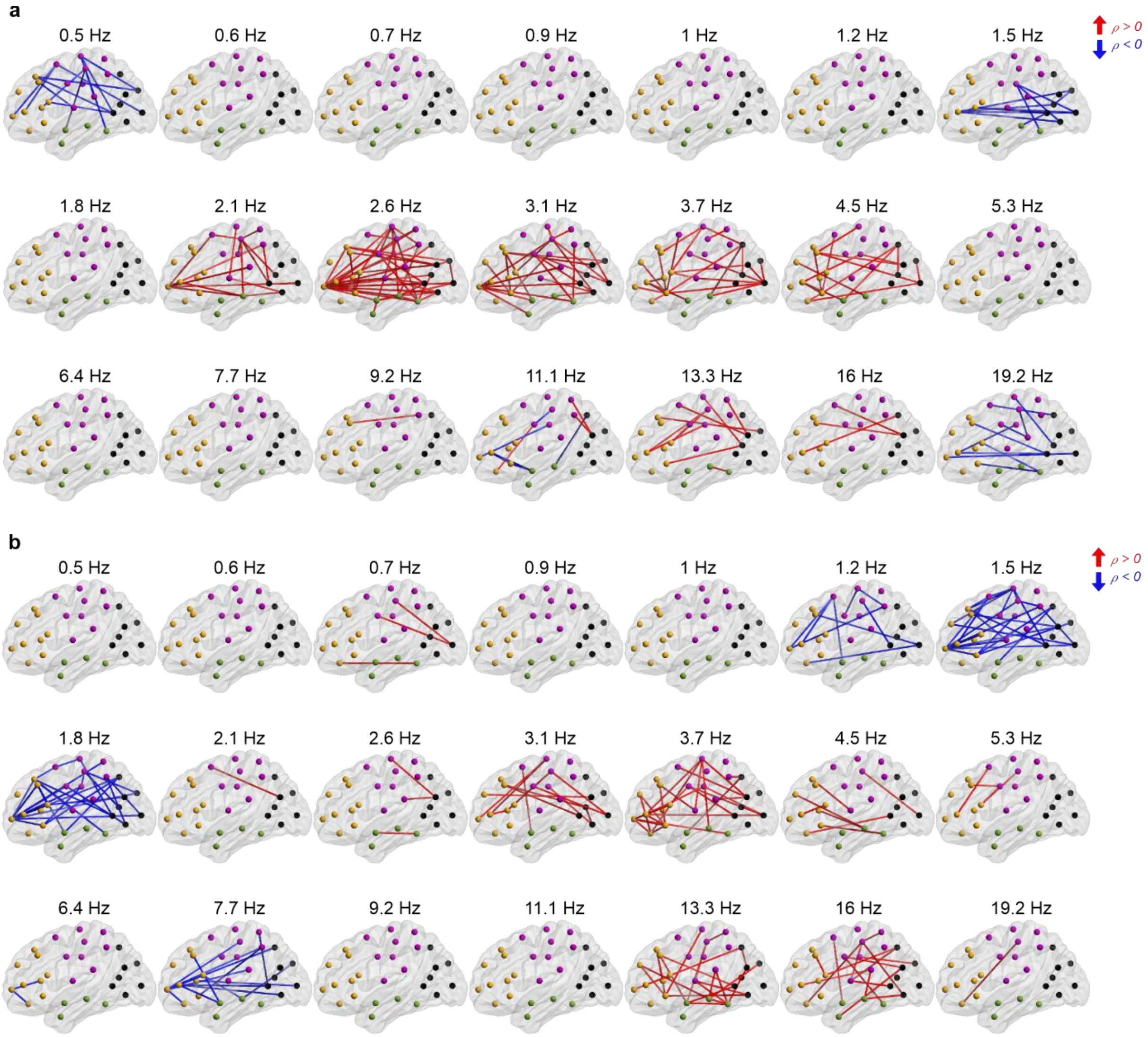

Figure 3—figure supplement 3. **The frequency-specific PPC correlation networks to C1 neurological outcomes.** 3-dimensional visualizations depicting the correlation of connection strength to C1 scores (Spearman, two-tailed test, with conceptional age as a covariate, and $\alpha = 0.05$) on all investigated frequency bands in AS (a) and QS (b) in EP infants at term-equivalent age. The edges displayed in the figure passed FDR correction ($q = 0.05$). Red networks indicate positive correlation (Spearman $\rho \geq 0$), whereas blue connections express negative correlation (Spearman $\rho < 0$).

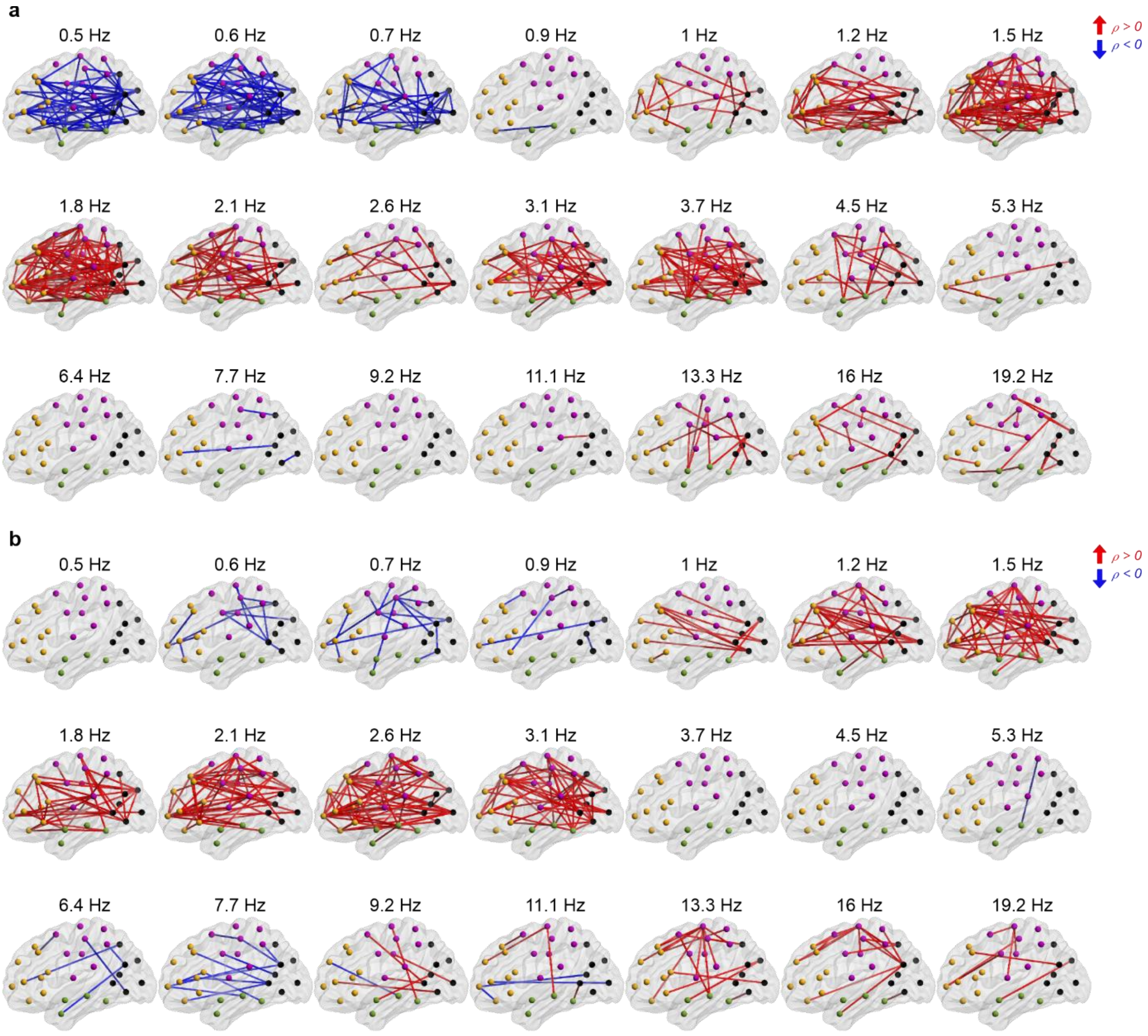

Figure 3—figure supplement 4. **The frequency-specific PPC correlation networks to C2 neurological outcomes.** 3-dimensional visualizations depicting the correlation of connection strength to C2 scores (Spearman, two-tailed test, with conceptional age as a covariate, and $\alpha = 0.05$) on all investigated frequency bands in AS (a) and QS (b) in EP infants at term-equivalent age. The edges displayed in the figure passed FDR correction ($q = 0.05$). Red networks indicate positive correlation (Spearman $\rho \geq 0$), whereas blue connections express negative correlation (Spearman $\rho < 0$).

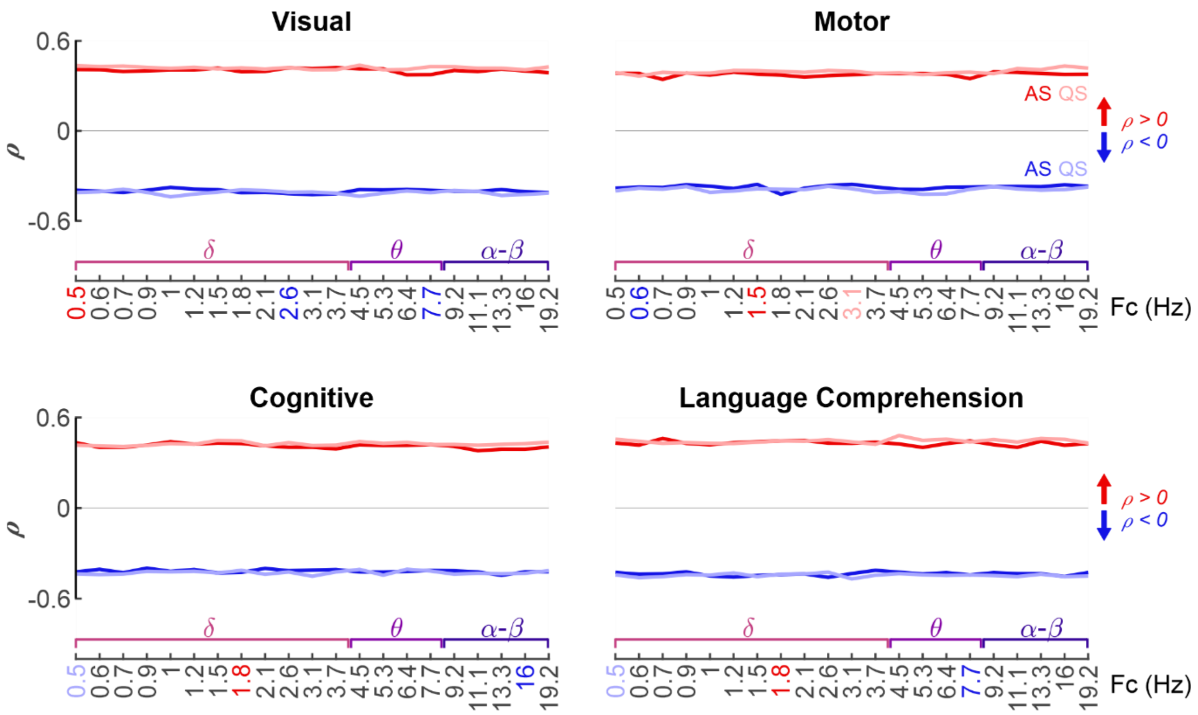

Figure 4—figure supplement 1. **Effect size of PPC networks depicting long-term neurocognitive correlation.** The effect size was computed as the mean Spearman (two-tailed test, with conceptional age as a covariate, and α = 0.05) ρ-value for the significant networks of each frequency band. The colours show the sign of the correlation ($\rho \geq 0$: red and $\rho < 0$: blue). The effect size values are presented separately for active sleep (AS, dark hues) and quiet sleep (QS, light hues) as well as for the neurocognitive scores Griffiths visual (upper left), Griffiths motor (upper right), Bayley cognitive (lower left), and Bayley language comprehension (lower right). The coloured numbers depict the frequencies with the most extensive correlations (Figure 4).

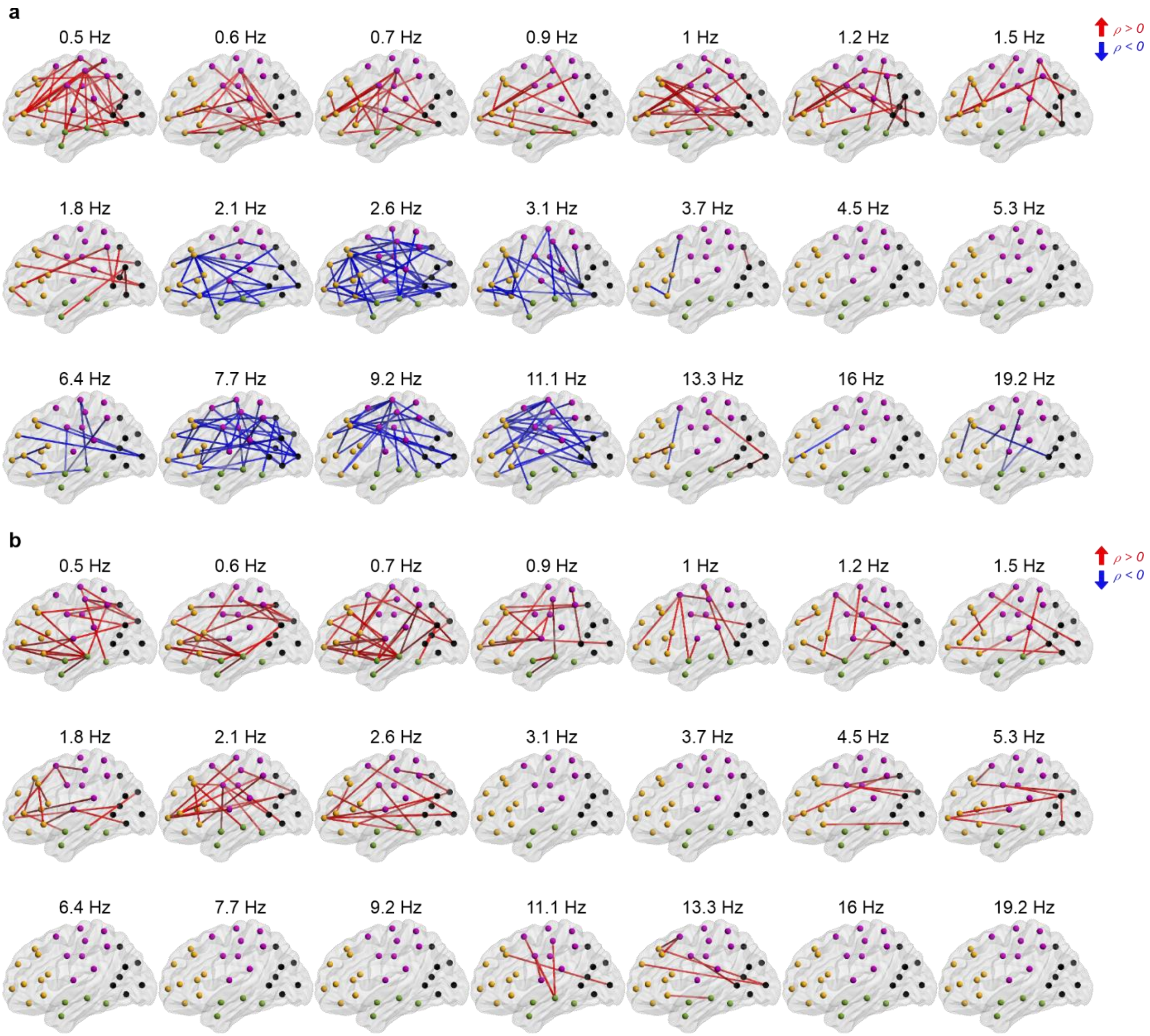

Figure 4—figure supplement 2. **The frequency-selective PPC fingerprint networks reflecting visual performance at 2 years of age.** Spatial visualizations of PPC edge strength correlation to Griffiths visual scores (Spearman, two-tailed test, with conceptional age as a covariate, $\alpha = 0.05$) over all examined frequency bands in AS (a) and QS (b) in the EP cohort at 2 years of age. The presented connections survived multiple comparisons correction with FDR ($q = 0.05$). Colour coding represents the sign of correlation (red: $\rho \geq 0$, blue $\rho < 0$).

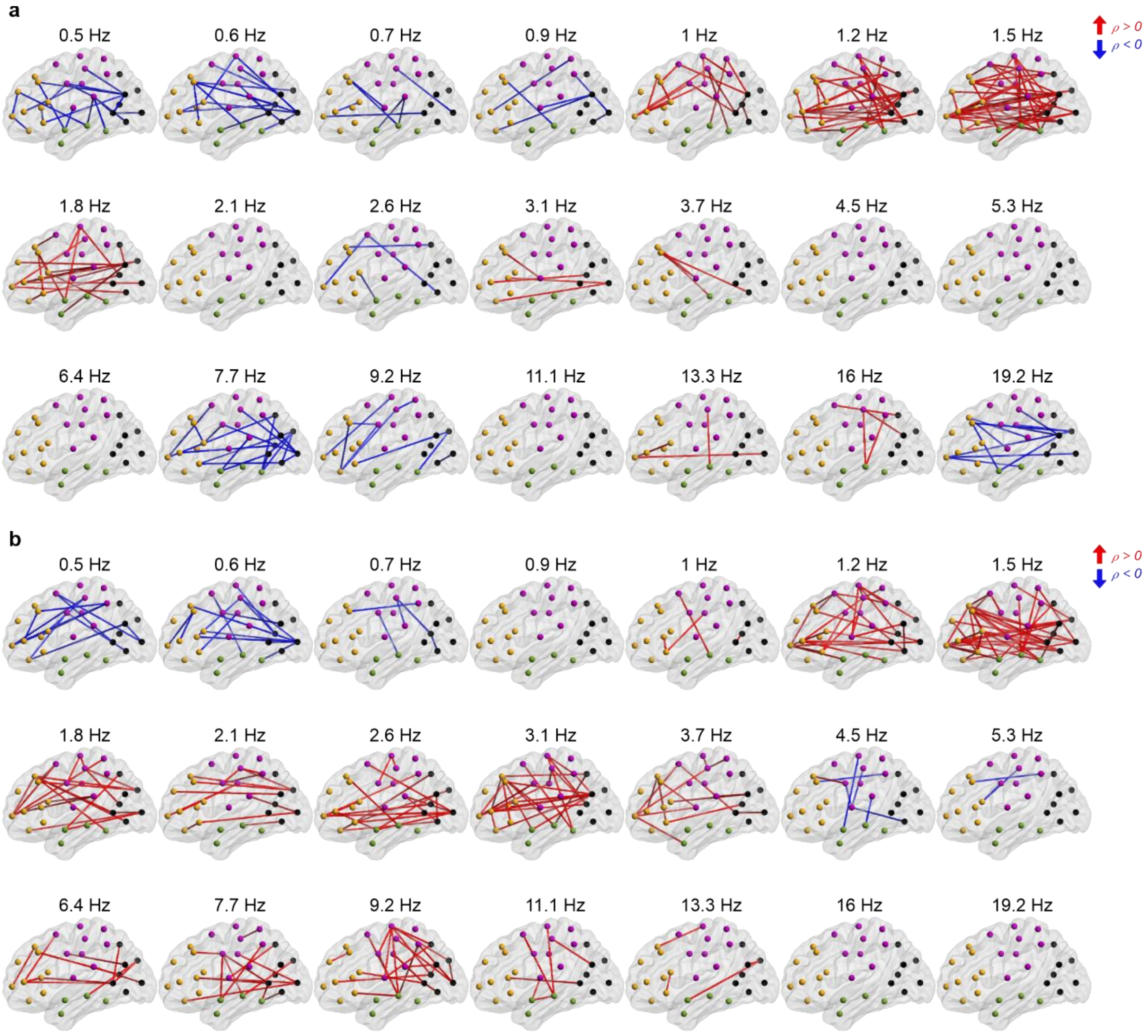

Figure 4—figure supplement 3. **The frequency-selective PPC fingerprint networks reflecting motor performance at 2 years of age.** Spatial visualizations of PPC edge strength correlation to Griffiths motor scores (Spearman, two-tailed test, with conceptional age as a covariate, $\alpha = 0.05$) over all examined frequency bands in AS (a) and QS (b) in the EP cohort at 2 years of age. The presented connections survived multiple comparisons correction with FDR ($q = 0.05$). Colour coding represents the sign of correlation (red: $\rho \geq 0$, blue $\rho < 0$).

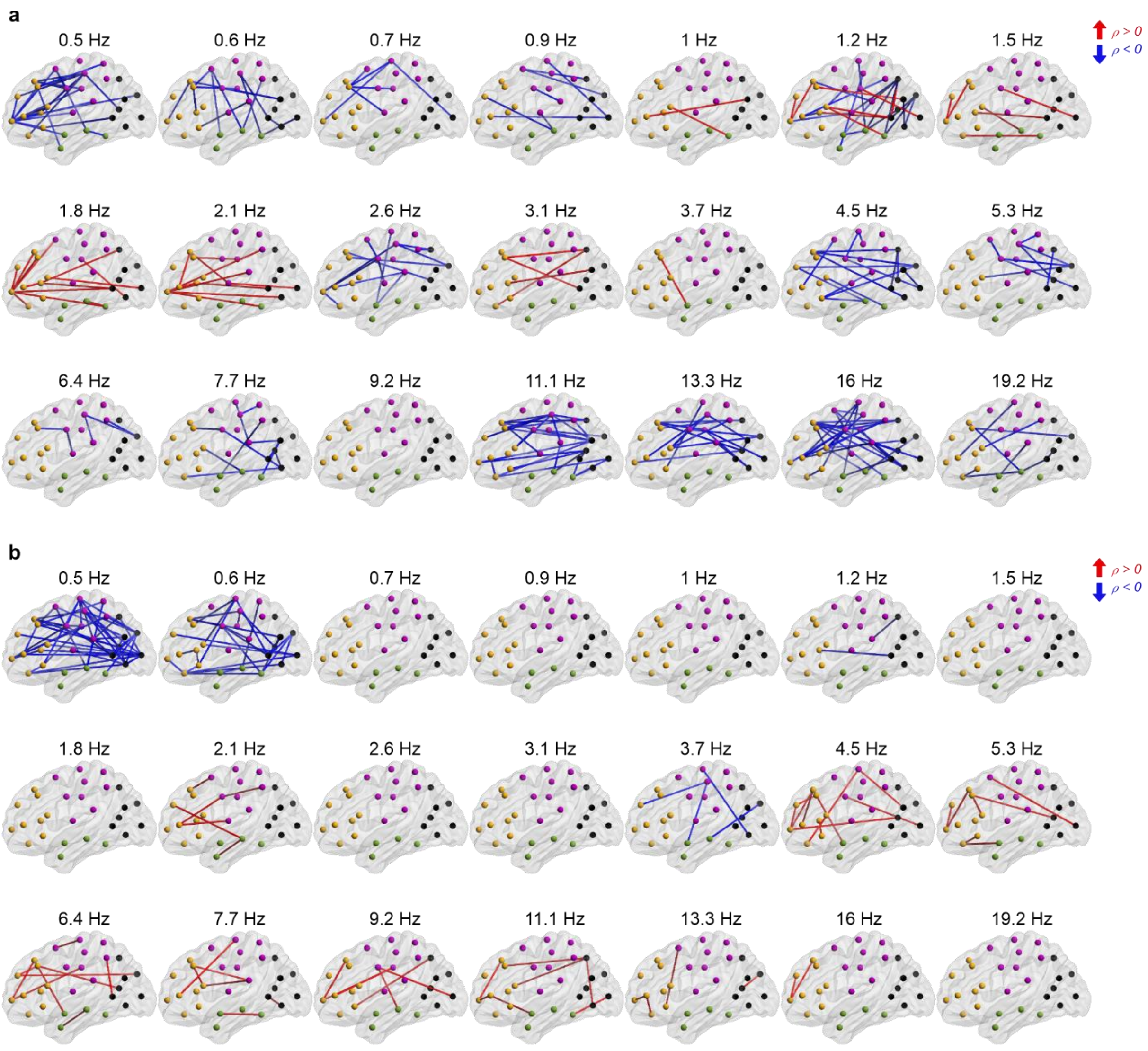

Figure 4—figure supplement 4. **The frequency-selective PPC fingerprint networks reflecting cognitive performance at 2 years of age.** Spatial visualizations of PPC edge strength correlation to Bayley cognitive scores (Spearman, two-tailed test, with conceptional age as a covariate, and 0.05) over all examined frequency bands in AS (a) and QS (b) in the EP cohort at 2 years of age. The presented connections survived multiple comparisons correction with FDR (q = 0.05). Colour coding represents the sign of correlation (red: $\rho \geq 0$, blue $\rho < 0$).

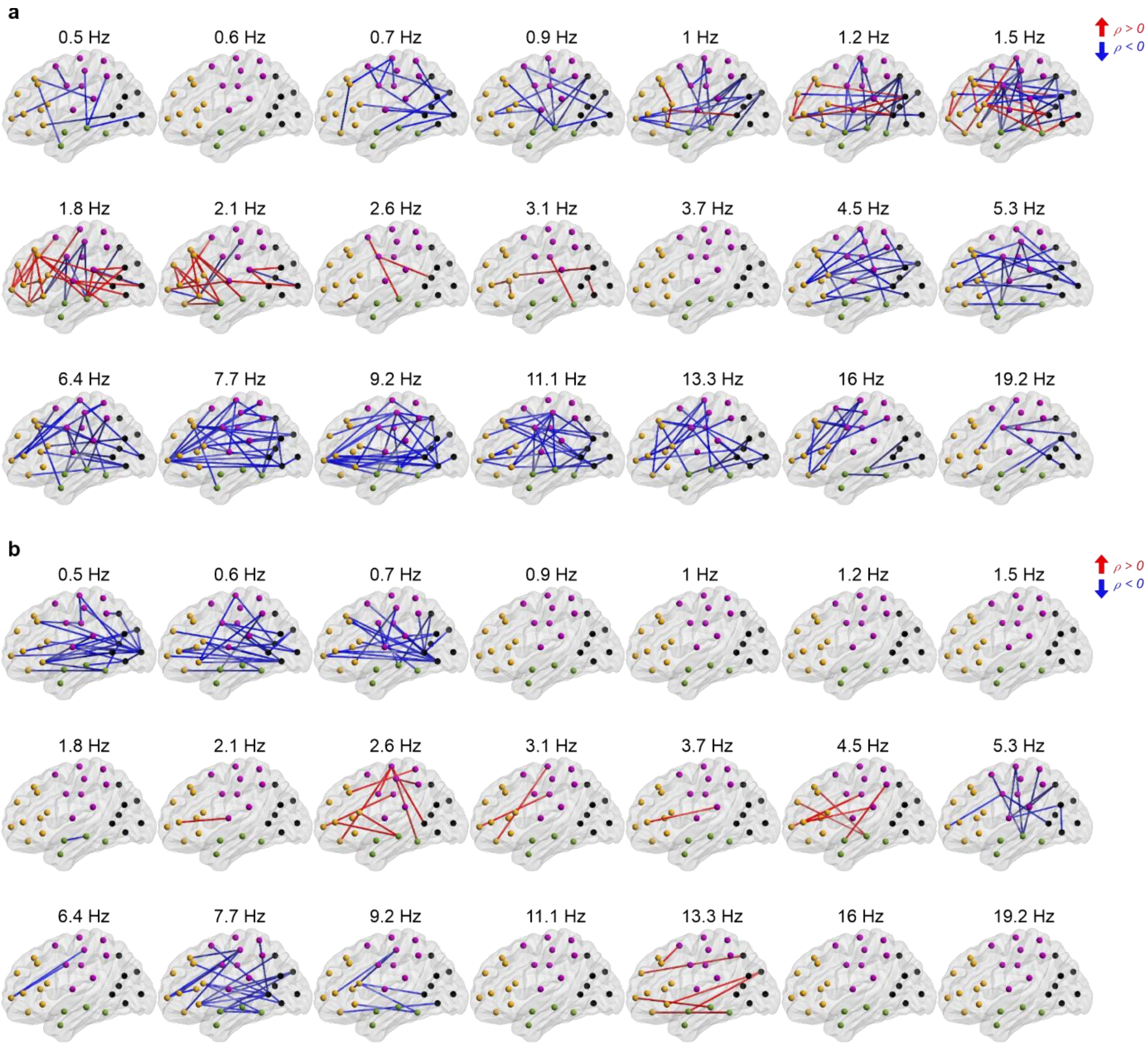

Figure 4—figure supplement 5. **The frequency-selective PPC fingerprint networks reflecting language comprehension at 2 years of age.** Spatial visualizations of PPC edge strength correlation to Bayley language comprehension scores (Spearman, two-tailed test, with conceptional age as a covariate, $\alpha = 0.05$) over all examined frequency bands in AS (a) and QS (b) in the EP cohort at 2 years of age. The presented connections survived multiple comparisons correction with FDR ($q = 0.05$). Colour coding represents the sign of correlation (red: $\rho \geq 0$, blue $\rho < 0$).
